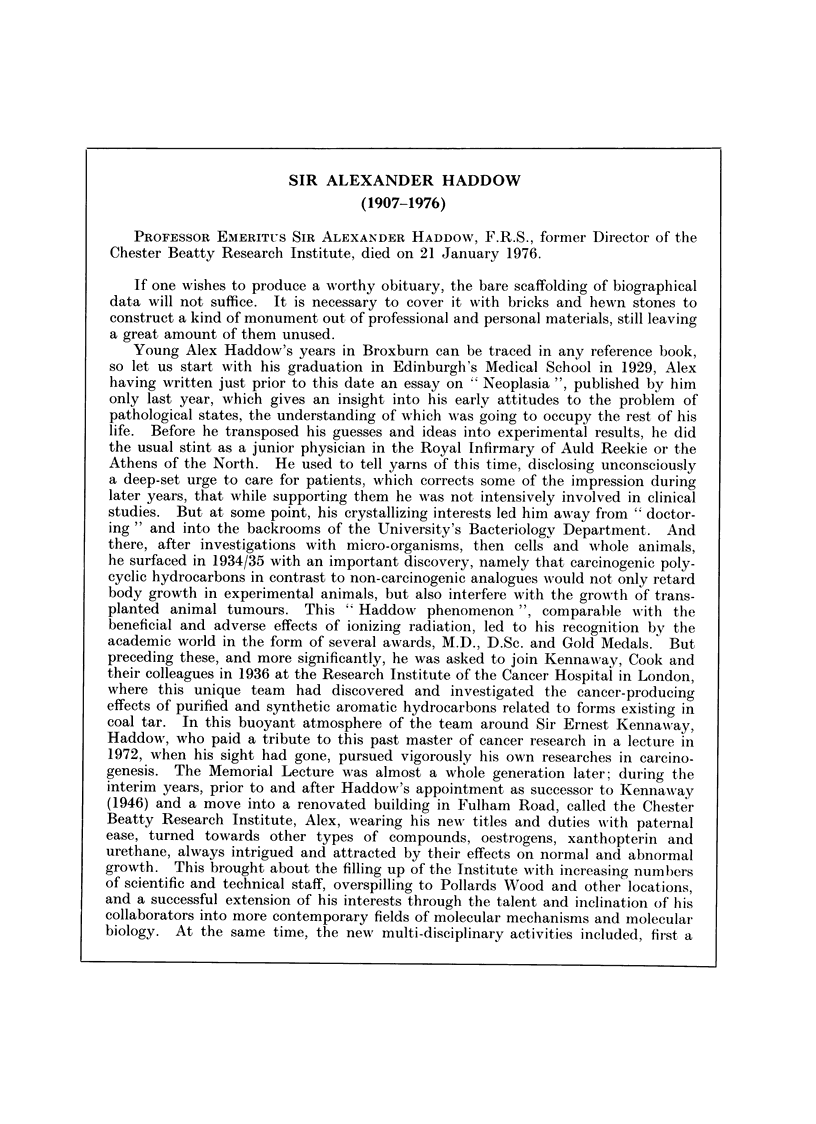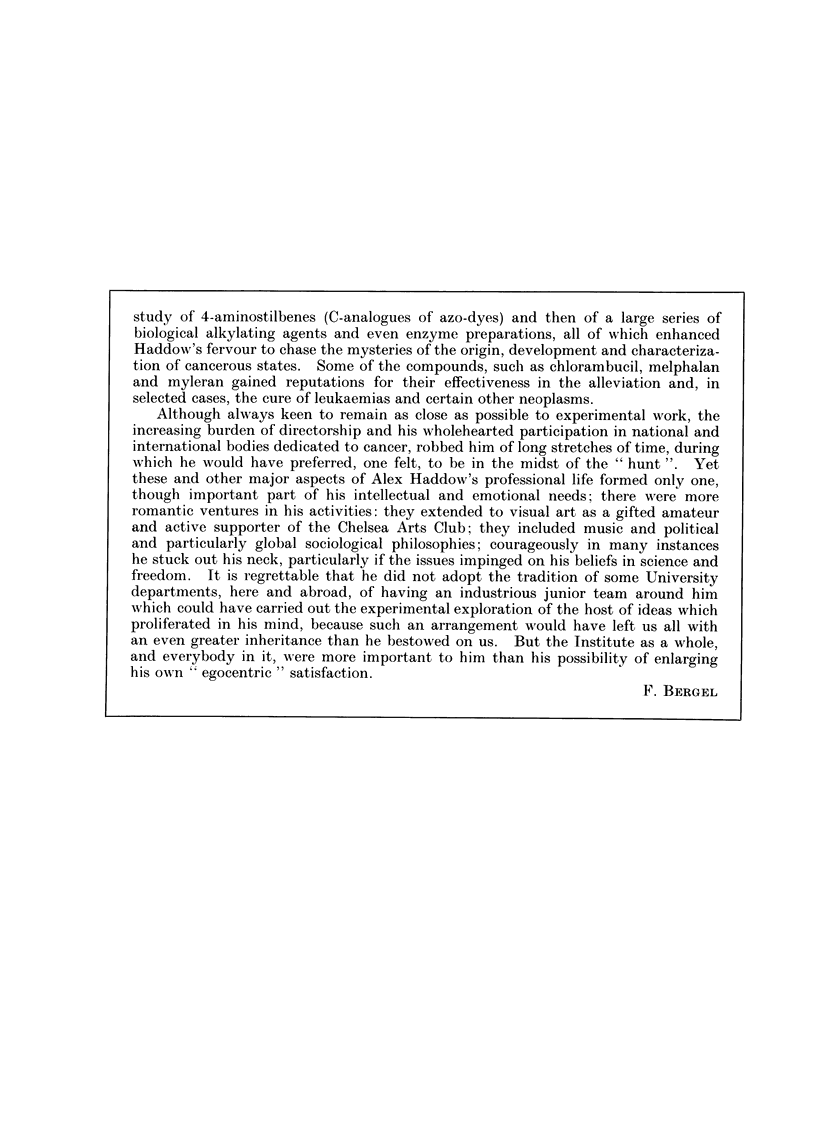# Sir Alexander Haddow (1907-1976)

**Published:** 1976-04

**Authors:** F. Bergel


### SIR ALEXANDER HADDOW

### (1907–1976)

PROFESSOR EMERITUS SIR ALEXANDER HADDOW, F.R.S., former Director of the Chester Beatty Research Institute, died on 21 January 1976.

If one wishes to produce a worthy obituary, the bare scaffolding of biographical data will not suffice. It is necessary to cover it with bricks and hewn stones to construct a kind of monument out of professional and personal materials, still leaving a great amount of them unused.

Young Alex Haddow's years in Broxburn can be traced in any reference book, so let us start with his graduation in Edinburgh's Medical School in 1929, Alex having written just prior to this date an essay on " Neoplasia ", published by him only last year, which gives an insight into his early attitudes to the problem of pathological states, the understanding of which was going to occupy the rest of his life. Before he transposed his guesses and ideas into experimental results, he did the usual stint as a junior physician in the Royal Infirmary of Auld Reekie or the Athens of the North. He used to tell yarns of this time, disclosing unconsciously a deep-set urge to care for patients, which corrects some of the impression during later years, that while supporting them he was not intensively involved in clinical studies. But at some point, his crystallizing interests led him away from " doctoring " and into the backrooms of the University's Bacteriology Department. And there, after investigations with micro-organisms, then cells and whole animals, he surfaced in 1934/35 with an important discovery, namely that carcinogenic polycyclic hydrocarbons in contrast to non-carcinogenic analogues would not only retard body growth in experimental animals, but also interfere with the growth of transplanted animal tumours. This " Haddow phenomenon ", comparable with the beneficial and adverse effects of ionizing radiation, led to his recognition by the academic world in the form of several awards, M.D., D.Sc. and Gold Medals. But preceding these, and more significantly, he was asked to join Kennaway, Cook and their colleagues in 1936 at the Research Institute of the Cancer Hospital in London, where this unique team had discovered and investigated the cancer-producing effects of purified and synthetic aromatic hydrocarbons related to forms existing in coal tar. In this buoyant atmosphere of the team around Sir Ernest Kennaway, Haddow, who paid a tribute to this past master of cancer research in a lecture in 1972, when his sight had gone, pursued vigorously his own researches in carcinogenesis. The Memorial Lecture was almost a whole generation later; during the interim years, prior to and after Haddow's appointment as successor to Kennaway (1946) and a move into a renovated building in Fulham Road, called the Chester Beatty Research Institute, Alex, wearing his new titles and duties with paternal ease, turned towards other types of compounds, oestrogens, xanthopterin and urethane, always intrigued and attracted by their effects on normal and abnormal growth. This brought about the filling up of the Institute with increasing numbers of scientific and technical staff, overspilling to Pollards Wood and other locations, and a successful extension of his interests through the talent and inclination of his collaborators into more contemporary fields of molecular mechanisms and molecular biology. At the same time, the new multi-disciplinary activities included, first a study of 4-aminostilbenes (C-analogues of azo-dyes) and then of a large series of biological alkylating agents and even enzyme preparations, all of which enhanced Haddow's fervour to chase the mysteries of the origin, development and characterization of cancerous states. Some of the compounds, such as chlorambucil, melphalan and myleran gained reputations for their effectiveness in the alleviation and, in selected cases, the cure of leukaemias and certain other neoplasms.

Although always keen to remain as close as possible to experimental work, the increasing burden of directorship and his wholehearted participation in national and international bodies dedicated to cancer, robbed him of long stretches of time, during which he would have preferred, one felt, to be in the midst of the " hunt ". Yet these and other major aspects of Alex Haddow's professional life formed only one, though important part of his intellectual and emotional needs; there were more romantic ventures in his activities: they extended to visual art as a gifted amateur and active supporter of the Chelsea Arts Club; they included music and political and particularly global sociological philosophies; courageously in many instances he stuck out his neck, particularly if the issues impinged on his beliefs in science and freedom. It is regrettable that he did not adopt the tradition of some University departments, here and abroad, of having an industrious junior team around him which could have carried out the experimental exploration of the host of ideas which proliferated in his mind, because such an arrangement would have left us all with an even greater inheritance than he bestowed on us. But the Institute as a whole, and everybody in it, were more important to him than his possibility of enlarging his own " egocentric " satisfaction.

F. BERGEL